# *N*′-(2,4-Dichloro­benzyl­idene)-4-methoxy­benzohydrazide methanol solvate

**DOI:** 10.1107/S1600536809022624

**Published:** 2009-06-17

**Authors:** Min Liang, Dong-Hui Zou

**Affiliations:** a College of Chemistry and Chemical Engineering, Qiqihar University, Qiqihar 161006, People’s Republic of China; b College of Life Science and Engineering, Qiqihar University, Qiqihar 161006, People’s Republic of China

## Abstract

In the title compound, C_15_H_12_Cl_2_N_2_O_2_·CH_3_OH, the hydrazone mol­ecule displays an *E* configuration about the C=N bond. The dihedral angle between the two benzene rings is 4.6 (2)°. In the crystal structure, the hydrazone and methanol mol­ecules are linked into a chain propagating along the *a* axis *via* N—H⋯O and O—H⋯O hydrogen bonds.

## Related literature

For the biological properties of hydrazone compounds, see: Küçükgüzel *et al.* (2003 ▶); Charkoudian *et al.* (2007 ▶). For the crystal structures of hydrazone compounds, see: Fun *et al.* (2008 ▶); Lo & Ng (2009 ▶); Ren (2009 ▶); Zhang (2009 ▶). For related structures, see: Wu (2009 ▶); Peng & Hou (2008 ▶); Mohd Lair *et al.* (2009 ▶).
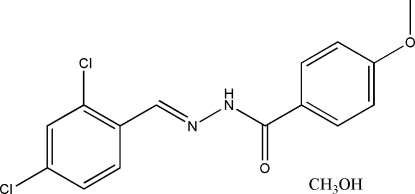


## Experimental

### 

#### Crystal data

C_15_H_12_Cl_2_N_2_O_2_·CH_4_O
*M* *_r_* = 355.21
Triclinic, 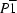

*a* = 6.7401 (11) Å
*b* = 8.9583 (14) Å
*c* = 14.567 (2) Å
α = 75.085 (2)°
β = 81.570 (2)°
γ = 83.445 (2)°
*V* = 838.1 (2) Å^3^
*Z* = 2
Mo *K*α radiation
μ = 0.40 mm^−1^
*T* = 298 K
0.20 × 0.18 × 0.18 mm

#### Data collection

Bruker SMART CCD area-detector diffractometer
Absorption correction: multi-scan (*SADABS*; Sheldrick, 1996 ▶) *T*_min_ = 0.924, *T*_max_ = 0.931
4896 measured reflections
3557 independent reflections
2461 reflections with *I* > 2σ(*I*)
*R*_int_ = 0.017

#### Refinement

*R*[*F* ^2^ > 2σ(*F* ^2^)] = 0.049
*wR*(*F* ^2^) = 0.128
*S* = 1.03
3557 reflections
214 parameters
1 restraint
H atoms treated by a mixture of independent and constrained refinement
Δρ_max_ = 0.20 e Å^−3^
Δρ_min_ = −0.31 e Å^−3^

### 

Data collection: *SMART* (Bruker, 1998 ▶); cell refinement: *SAINT* (Bruker, 1998 ▶); data reduction: *SAINT*; program(s) used to solve structure: *SHELXS97* (Sheldrick, 2008 ▶); program(s) used to refine structure: *SHELXL97* (Sheldrick, 2008 ▶); molecular graphics: *SHELXTL* (Sheldrick, 2008 ▶); software used to prepare material for publication: *SHELXTL*.

## Supplementary Material

Crystal structure: contains datablocks global, I. DOI: 10.1107/S1600536809022624/ci2826sup1.cif

Structure factors: contains datablocks I. DOI: 10.1107/S1600536809022624/ci2826Isup2.hkl

Additional supplementary materials:  crystallographic information; 3D view; checkCIF report

## Figures and Tables

**Table 1:** Hydrogen-bond geometry (Å, °)

*D*—H⋯*A*	*D*—H	H⋯*A*	*D*⋯*A*	*D*—H⋯*A*
N2—H2⋯O3^i^	0.893 (10)	2.013 (12)	2.889 (3)	167 (3)
O3—H3⋯O1^ii^	0.82	1.99	2.780 (2)	163

Symmetry codes: (i) 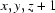; (ii) 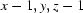.

**Fig. 1.**
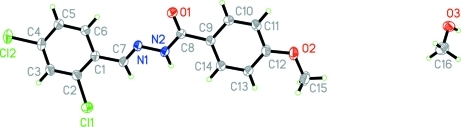
The asymmetric unit of the title compound, showing 30% probability displacement ellipsoids for the non-H atoms. H atoms are shown as spheres of arbitrary radius.

**Fig. 2.**
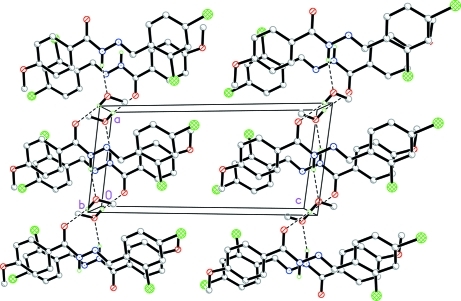
The packing diagram, viewed along the b axis. H atoms not involved in hydrogen bonding (dashed lines) have been omitted for clarity.

## supplementary crystallographic information

### Comment

Hydrazones possess excellent antibacterial, antifungal, and antitumor activities (Küçükgüzel *et al.*, 2003; Charkoudian *et al.*, 2007). Recently, the crystal structures of some hydrazone compounds have been reported (Fun *et al.*, 2008; Lo & Ng, 2009; Ren, 2009; Zhang, 2009). We report herein the crystal structure of the title new hydrazone compound.

The asymmetric unit of the title compound contains a hydrazone molecule and a methanol molecule. In the hydrazone molecule, the dihedral angle between the two benzene rings is 4.6 (2)°. The hydrazone molecule exists in an E configuration with respect to the methylidene group. All the bond lengths are normal and comparable to those in similar hydrazone compounds (Wu, 2009; Peng & Hou, 2008; Mohd Lair *et al.*, 2009).

In the crystal structure of the title compound, the hydrazone molecules are linked by the methanol molecules through N—H···O and O—H···O hydrogen bonds (Table 1), forming chains propagating along the *a* axis (Fig. 2).

### Experimental

Equimolar quantities (1.0 mmol each) of 2,4-dichlorobenzaldehyde and 4-methoxybenzohydrazide were mixed and refluxed in methanol. The reaction mixture was cooled to room temperature to give a clear colourless solution. Colourless single crystals of the title compound were formed by slow evaporation of the solution in air.

### Refinement

Atom H2 was located in a difference map and refined isotropically, with the N—H distance restrained to 0.90 (1) Å. Other H atoms were placed in calculated positions (C—H = 0.93–0.96 Å and O—H = 0.82 Å) and refined as riding with *U*_iso_(H) = 1.2*U*_eq_(C) and 1.5*U*_eq_(O,C_methyl_).

### Crystal data

**Table:**

C_15_H_12_Cl_2_N_2_O_2_·CH_4_O	*Z* = 2
*M**_r_* = 355.21	*F*(000) = 368
Triclinic, *P*1	*D*_x_ = 1.408 Mg m^−^^3^
Hall symbol: -P 1	Mo *K*α radiation, λ = 0.71073 Å
*a* = 6.7401 (11) Å	Cell parameters from 1307 reflections
*b* = 8.9583 (14) Å	θ = 2.3–26.7°
*c* = 14.567 (2) Å	µ = 0.40 mm^−^^1^
α = 75.085 (2)°	*T* = 298 K
β = 81.570 (2)°	Block, colourless
γ = 83.445 (2)°	0.20 × 0.18 × 0.18 mm
*V* = 838.1 (2) Å^3^	

### Data collection

**Table:**

Bruker SMART CCD area-detector diffractometer	3557 independent reflections
Radiation source: fine-focus sealed tube	2461 reflections with *I* > 2σ(*I*)
graphite	*R*_int_ = 0.017
ω scans	θ_max_ = 27.0°, θ_min_ = 2.4°
Absorption correction: multi-scan (*SADABS*; Sheldrick, 1996)	*h* = −8→8
*T*_min_ = 0.924, *T*_max_ = 0.931	*k* = −11→11
4896 measured reflections	*l* = −18→16

### Refinement

**Table:**

Refinement on *F*^2^	Primary atom site location: structure-invariant direct methods
Least-squares matrix: full	Secondary atom site location: difference Fourier map
*R*[*F*^2^ > 2σ(*F*^2^)] = 0.049	Hydrogen site location: inferred from neighbouring sites
*wR*(*F*^2^) = 0.128	H atoms treated by a mixture of independent and constrained refinement
*S* = 1.03	*w* = 1/[σ^2^(*F*_o_^2^) + (0.048*P*)^2^ + 0.3404*P*] where *P* = (*F*_o_^2^ + 2*F*_c_^2^)/3
3557 reflections	(Δ/σ)_max_ = 0.001
214 parameters	Δρ_max_ = 0.20 e Å^−^^3^
1 restraint	Δρ_min_ = −0.31 e Å^−^^3^

### Special details

**Table:**

Geometry. All e.s.d.'s (except the e.s.d. in the dihedral angle between two l.s. planes) are estimated using the full covariance matrix. The cell e.s.d.'s are taken into account individually in the estimation of e.s.d.'s in distances, angles and torsion angles; correlations between e.s.d.'s in cell parameters are only used when they are defined by crystal symmetry. An approximate (isotropic) treatment of cell e.s.d.'s is used for estimating e.s.d.'s involving l.s. planes.
Refinement. Refinement of *F*^2^ against ALL reflections. The weighted *R*-factor *wR* and goodness of fit *S* are based on *F*^2^, conventional *R*-factors *R* are based on *F*, with *F* set to zero for negative *F*^2^. The threshold expression of *F*^2^ > σ(*F*^2^) is used only for calculating *R*-factors(gt) *etc*. and is not relevant to the choice of reflections for refinement. *R*-factors based on *F*^2^ are statistically about twice as large as those based on *F*, and *R*- factors based on ALL data will be even larger.

### Fractional atomic coordinates and isotropic or equivalent isotropic displacement parameters (Å^2^)

**Table:**

	*x*	*y*	*z*	*U*_iso_*/*U*_eq_	
Cl1	0.24871 (10)	0.73959 (10)	1.31319 (5)	0.0692 (2)	
Cl2	0.81448 (12)	0.99939 (9)	1.42996 (5)	0.0719 (3)	
N1	0.6168 (3)	0.8075 (2)	1.04117 (13)	0.0442 (5)	
N2	0.5388 (3)	0.7708 (2)	0.96816 (13)	0.0451 (5)	
O1	0.8442 (2)	0.7876 (2)	0.88034 (12)	0.0582 (5)	
O2	0.4029 (3)	0.5972 (2)	0.58129 (13)	0.0661 (5)	
O3	0.1098 (3)	0.7492 (2)	0.01521 (13)	0.0613 (5)	
H3	0.0183	0.7737	−0.0185	0.092*	
C1	0.5714 (3)	0.8617 (3)	1.19309 (16)	0.0415 (5)	
C2	0.4710 (3)	0.8331 (3)	1.28613 (17)	0.0451 (6)	
C3	0.5433 (4)	0.8734 (3)	1.35947 (17)	0.0502 (6)	
H3A	0.4748	0.8523	1.4210	0.060*	
C4	0.7199 (4)	0.9456 (3)	1.33905 (17)	0.0501 (6)	
C5	0.8244 (4)	0.9770 (3)	1.24829 (17)	0.0506 (6)	
H5	0.9432	1.0262	1.2357	0.061*	
C6	0.7498 (4)	0.9343 (3)	1.17690 (17)	0.0469 (6)	
H6	0.8205	0.9544	1.1158	0.056*	
C7	0.4950 (4)	0.8199 (3)	1.11445 (16)	0.0462 (6)	
H7	0.3601	0.8029	1.1183	0.055*	
C8	0.6669 (3)	0.7618 (3)	0.88846 (16)	0.0416 (5)	
C9	0.5841 (3)	0.7173 (3)	0.81095 (15)	0.0411 (5)	
C10	0.6995 (4)	0.7383 (3)	0.72207 (17)	0.0490 (6)	
H10	0.8226	0.7809	0.7130	0.059*	
C11	0.6354 (4)	0.6974 (3)	0.64766 (17)	0.0553 (7)	
H11	0.7148	0.7129	0.5887	0.066*	
C12	0.4530 (4)	0.6333 (3)	0.65966 (17)	0.0487 (6)	
C13	0.3364 (4)	0.6112 (3)	0.74703 (18)	0.0557 (7)	
H13	0.2135	0.5685	0.7559	0.067*	
C14	0.4030 (4)	0.6528 (3)	0.82198 (17)	0.0542 (7)	
H14	0.3238	0.6370	0.8810	0.065*	
C15	0.2166 (5)	0.5339 (4)	0.5880 (2)	0.0752 (9)	
H15A	0.2099	0.4415	0.6391	0.113*	
H15B	0.2050	0.5092	0.5288	0.113*	
H15C	0.1084	0.6081	0.6008	0.113*	
C16	0.0604 (5)	0.6214 (3)	0.0907 (2)	0.0715 (8)	
H16A	0.1791	0.5753	0.1196	0.107*	
H16B	0.0061	0.5466	0.0666	0.107*	
H16C	−0.0377	0.6547	0.1376	0.107*	
H2	0.4070 (17)	0.761 (3)	0.973 (2)	0.080*	

### Atomic displacement parameters (Å^2^)

**Table:**

	*U*^11^	*U*^22^	*U*^33^	*U*^12^	*U*^13^	*U*^23^
Cl1	0.0488 (4)	0.1016 (6)	0.0583 (4)	−0.0229 (4)	0.0003 (3)	−0.0181 (4)
Cl2	0.0865 (5)	0.0888 (6)	0.0534 (4)	−0.0131 (4)	−0.0237 (4)	−0.0297 (4)
N1	0.0427 (11)	0.0564 (12)	0.0384 (10)	−0.0082 (9)	−0.0097 (8)	−0.0159 (9)
N2	0.0362 (10)	0.0655 (13)	0.0396 (10)	−0.0100 (10)	−0.0066 (8)	−0.0202 (9)
O1	0.0410 (10)	0.0925 (14)	0.0470 (10)	−0.0223 (9)	−0.0023 (7)	−0.0221 (9)
O2	0.0677 (12)	0.0936 (15)	0.0495 (11)	−0.0154 (11)	−0.0066 (9)	−0.0365 (10)
O3	0.0389 (10)	0.0899 (14)	0.0539 (11)	−0.0146 (9)	−0.0084 (8)	−0.0097 (10)
C1	0.0419 (13)	0.0438 (13)	0.0402 (12)	−0.0003 (10)	−0.0089 (10)	−0.0120 (10)
C2	0.0386 (12)	0.0523 (15)	0.0439 (13)	−0.0012 (11)	−0.0064 (10)	−0.0114 (11)
C3	0.0509 (15)	0.0626 (16)	0.0381 (13)	−0.0006 (12)	−0.0054 (10)	−0.0158 (11)
C4	0.0601 (16)	0.0531 (15)	0.0432 (13)	0.0014 (12)	−0.0188 (11)	−0.0183 (11)
C5	0.0531 (15)	0.0544 (15)	0.0494 (14)	−0.0124 (12)	−0.0117 (11)	−0.0152 (12)
C6	0.0495 (14)	0.0539 (15)	0.0386 (12)	−0.0110 (11)	−0.0029 (10)	−0.0121 (11)
C7	0.0399 (13)	0.0586 (15)	0.0442 (13)	−0.0076 (11)	−0.0088 (10)	−0.0164 (11)
C8	0.0399 (13)	0.0474 (14)	0.0384 (12)	−0.0083 (10)	−0.0066 (10)	−0.0089 (10)
C9	0.0392 (12)	0.0472 (13)	0.0384 (12)	−0.0056 (10)	−0.0057 (9)	−0.0116 (10)
C10	0.0406 (13)	0.0641 (16)	0.0443 (13)	−0.0132 (11)	−0.0016 (10)	−0.0151 (12)
C11	0.0531 (15)	0.0764 (19)	0.0391 (13)	−0.0106 (13)	0.0034 (11)	−0.0216 (13)
C12	0.0527 (15)	0.0576 (15)	0.0411 (13)	−0.0039 (12)	−0.0089 (11)	−0.0198 (11)
C13	0.0486 (15)	0.0778 (19)	0.0495 (14)	−0.0240 (13)	−0.0027 (11)	−0.0251 (13)
C14	0.0519 (15)	0.0768 (18)	0.0394 (13)	−0.0224 (13)	0.0029 (11)	−0.0215 (12)
C15	0.071 (2)	0.102 (2)	0.0701 (19)	−0.0163 (17)	−0.0211 (16)	−0.0415 (18)
C16	0.0693 (19)	0.0626 (19)	0.084 (2)	−0.0067 (15)	−0.0218 (16)	−0.0125 (16)

### Geometric parameters (Å, °)

**Table:**

Cl1—C2	1.745 (2)	C6—H6	0.93
Cl2—C4	1.743 (2)	C7—H7	0.93
N1—C7	1.268 (3)	C8—C9	1.487 (3)
N1—N2	1.378 (2)	C9—C14	1.380 (3)
N2—C8	1.356 (3)	C9—C10	1.389 (3)
N2—H2	0.893 (10)	C10—C11	1.369 (3)
O1—C8	1.226 (3)	C10—H10	0.93
O2—C12	1.360 (3)	C11—C12	1.385 (3)
O2—C15	1.417 (3)	C11—H11	0.93
O3—C16	1.401 (3)	C12—C13	1.374 (3)
O3—H3	0.82	C13—C14	1.387 (3)
C1—C6	1.392 (3)	C13—H13	0.93
C1—C2	1.397 (3)	C14—H14	0.93
C1—C7	1.468 (3)	C15—H15A	0.96
C2—C3	1.378 (3)	C15—H15B	0.96
C3—C4	1.377 (4)	C15—H15C	0.96
C3—H3A	0.93	C16—H16A	0.96
C4—C5	1.380 (3)	C16—H16B	0.96
C5—C6	1.373 (3)	C16—H16C	0.96
C5—H5	0.93		
			
C7—N1—N2	116.93 (19)	C14—C9—C10	117.8 (2)
C8—N2—N1	117.23 (18)	C14—C9—C8	124.5 (2)
C8—N2—H2	123.2 (19)	C10—C9—C8	117.7 (2)
N1—N2—H2	119.4 (19)	C11—C10—C9	121.2 (2)
C12—O2—C15	118.8 (2)	C11—C10—H10	119.4
C16—O3—H3	109.5	C9—C10—H10	119.4
C6—C1—C2	116.9 (2)	C10—C11—C12	120.4 (2)
C6—C1—C7	120.4 (2)	C10—C11—H11	119.8
C2—C1—C7	122.7 (2)	C12—C11—H11	119.8
C3—C2—C1	122.3 (2)	O2—C12—C13	124.8 (2)
C3—C2—Cl1	117.74 (19)	O2—C12—C11	115.8 (2)
C1—C2—Cl1	119.93 (17)	C13—C12—C11	119.4 (2)
C4—C3—C2	118.2 (2)	C12—C13—C14	119.7 (2)
C4—C3—H3A	120.9	C12—C13—H13	120.2
C2—C3—H3A	120.9	C14—C13—H13	120.2
C3—C4—C5	121.8 (2)	C9—C14—C13	121.5 (2)
C3—C4—Cl2	119.34 (19)	C9—C14—H14	119.2
C5—C4—Cl2	118.9 (2)	C13—C14—H14	119.2
C6—C5—C4	118.7 (2)	O2—C15—H15A	109.5
C6—C5—H5	120.6	O2—C15—H15B	109.5
C4—C5—H5	120.6	H15A—C15—H15B	109.5
C5—C6—C1	122.1 (2)	O2—C15—H15C	109.5
C5—C6—H6	119.0	H15A—C15—H15C	109.5
C1—C6—H6	119.0	H15B—C15—H15C	109.5
N1—C7—C1	118.6 (2)	O3—C16—H16A	109.5
N1—C7—H7	120.7	O3—C16—H16B	109.5
C1—C7—H7	120.7	H16A—C16—H16B	109.5
O1—C8—N2	121.9 (2)	O3—C16—H16C	109.5
O1—C8—C9	120.9 (2)	H16A—C16—H16C	109.5
N2—C8—C9	117.20 (19)	H16B—C16—H16C	109.5

### Hydrogen-bond geometry (Å, °)

**Table:**

*D*—H···*A*	*D*—H	H···*A*	*D*···*A*	*D*—H···*A*
N2—H2···O3^i^	0.89 (1)	2.01 (1)	2.889 (3)	167 (3)
O3—H3···O1^ii^	0.82	1.99	2.780 (2)	163

Symmetry codes: (i) *x*, *y*, *z*+1; (ii) *x*−1, *y*, *z*−1.
